# Noninvasive and safe cell viability assay for *Paramecium* using natural pigment extracted from food

**DOI:** 10.1038/s41598-020-67712-0

**Published:** 2020-07-03

**Authors:** Kyohei Yamashita, Eiji Tokunaga

**Affiliations:** Department of Physics, Faculty of Science, Tokyo University of Science, 1-3 Kagurazaka, Shinjuku-ku, Tokyo, 162-8601 Japan

**Keywords:** Biochemistry, Biological techniques, Biophysics, Biotechnology, Cell biology, Chemical biology, Drug discovery, Microbiology, Plant sciences, Environmental sciences, Medical research, Optics and photonics

## Abstract

Noninvasive, safe and cost-effective cell viability assay is important in many fields of biological research such as cell culture and counting. We examined ten typical natural pigments extracted from food to find that *Monascus* pigment (MP) or anthocyanin pigment (AP: purple sweet potato and purple cabbage) with Tris (Trimethylolaminomethane) works as a good indicator of viability assay for dye exclusion test (DET) of *Paramecium*. This was confirmed spectrally by scan-free, non-invasive absorbance spectral imaging *A* (*x*,* y*, *λ*) microscopy. We developed a new method of cell capture using a metal mesh to confine live *Paramecium* in a restricted space. This has the advantage that a low-cost and robust capture can be fabricated without using special equipment, compared to a conventional lab-on-a-chip. As a result, MP and AP stained dead cells as quick as methylene blue (MB), a synthetic dye conventionally used in DET within 1 min when treated with microwave and benzalkonium chloride. The natural pigments with Tris had little effect on inhibiting the growth of *Paramecium*, but MB killed all the cells within 1 h. MP is most useful because it allows non-invasive DET without Tris. This approach provides less invasive and safe DET.

## Introduction

*Paramecium* is a motile unicellular eukaryote whose cell has a long axis of 180–200 μm, a short axis of 20–30 μL, and a volume of 400 pL^1^. It is easy to culture, and has the advantage of growing in a shorter time compared to higher animals such as rats and mice^2^. Since *Paramecium* is a eukaryotic organism, *Paramecium* has been so far used as an indicator for toxicity tests against natural food pigments^2^, tar-based synthetic dyes^3^, caffeine^4^, and nanoparticles (cobalt ferrite, titanium oxide, Silver and carbon nanotubes)^1^. In addition, plasmids containing specific genes can be injected into the nucleus to induce transformation 24 h later^5^.

For the development of these technologies, non-invasive and safe cell viability assays play an important role as basic technologies. For example, there is a report that states "Death was assumed to have occurred when there was no movement of *Paramecium*^3^." However, there are some cells that are actually alive but do not show movement. When cell movement is not observed, viability can be determined from the presence or absence of cilia movement. This needs increased magnification for observation, reducing efficiency to count the cells due to a restricted field of view. In addition, since the production of mutants of *Paramecium* is subjected to significant physical and chemical burdens on the cells, it is necessary to confirm whether the cells are viable or dead in the screening.

The following methods are known as conventional methods for distinguishing between live and dead cells^6^. Dye exclusion test (DET) is a method to judge a cell stained with a synthetic dye such as trypan blue (TB) as a dead cell^7^. The colony formation assay evaluates the number of live cells by inoculating the diluted cell suspension on an agar culture and counting the number of colonies formed^8^. Enzyme activity assays estimate viability by the enzymatic reaction of enzymes in living cells or enzymes leaking from dead cells^9^. Flow cytometry analysis detects dead cells labeled with a fluorescent dye^10^ by fluorescence flow cytometry^11,12^. There is also an optical method where the dead or alive state of cells is diagnosed by deflection change of the probe light beam^13^.

However, these methods have drawbacks such as requiring special techniques and equipment, damaging cells, and inability to perform in situ measurements in a culturing process over time. In order to solve these problems, we propose a method for determining cell viability using natural food pigments, focusing on DET described above.

Methylene blue (MB) and TB, which are widely used as dyes of viability assay, have been used for DET. MB is often used to distinguish between dead and live cells^14^. However, the DET method with MB may suffer false positive results with longer exposure times^15^. TB is a diazo dye that is widely used to selectively color dead tissue or cells. The mechanism for staining cells by TB prevents its uptake into living cells with negatively charged membranes. Therefore, live cells are not stained, but dead cells with damaged cell membranes are stained with TB^16^. However, TB is known to cause environmental and cellular health problems due to its potential teratogenic effects^17,18^. It has also been pointed out that pore formation may be induced in the cell membrane in order to increase membrane permeability. As other dyes for the DET, eosin^19^, amethyst violet^20^, and nile blue^21^ have been used but it is known that the selective permeability of the plasma membrane is destroyed or severely impaired^7^, indicating that these dyes are toxic for cells.

To circumvent these problems, a technique was developed to count cells on a cell counter using erythrosine B (EB, also known as Red No. 3), which is used as a food additive^22^. This synthetic colorant is a food dye that does not pass through biological membranes and is compatible with automatic cell counters. However, because EB is suspected to be carcinogenic, FAD (Food and Drug Administration) has banned it for a period of time (1990)^23,24^. Recently, consumers have become more conscious of the ingredients of food, so that foods are required to be as “natural” as possible^25,26^. Therefore, research on food pigment extraction methods and their application to foods is underway^27^. In addition, a method has been developed to evaluate the cell staining properties of biological staining by observation under a multiphoton laser microscope^28^. The pigments used there include not only synthetic colorants but also natural food pigments. However, since a multiphoton laser microscope is used, cost and skill are required, and it cannot be said that it is for general purpose.

Therefore, when using natural food pigments that can be visually assessed at lower cost for DET than the methods that require expensive equipment, the pigments may not only reduce the burden on the cells in basic viability assays, but may also be widely used industrially. In this study, 10 natural pigments extracted from food and 2 traditional synthetic dyes for viability assay are tested, among which *Monascus* pigment (MP) and anthocyanin pigments (APs: purple sweet potato (PS) and purple cabbage (PC)) are of particular interest.

MP is derived from *Monascus* sp*.* This is a type of filamentous fungus. In MP, six major pigment components are known (monascorubrin, monascorubramine, rubropunctatin, rubropunctamine, monascin, ankaflavin), and their chemical structures have been elucidated^29−34^. The major pigment components of edible *monascus* pigments distributed in Japan are defined as ankaflavins and monascorubrins by the Japan’s Specifications and Standards for Food Additives (JSFA)^35^. The MP used in this study has a red color, suggesting that monascorburins are the main components. The color has little pH dependence (however, it tends to precipitate with acidic solutions), is relatively stable to heat, and has excellent staining for proteins^36^. On the other hand, it is unstable to light irradiation, especially under acidic conditions. MP is an inexpensive and reproducible substrate with color variations, high safety, and good solubility in water and ethanol^32,37^.

AP is a water-soluble pigment that dissolves as a glycoside in the vacuole of plants and has many chemical structures depending on the type of sugar and the organic acid that binds to it^36^. AP turns red under acidic conditions and turns blue as pH increases. Many APs are more stable under acidic conditions than basic conditions^38^. In particular, AP contained in purple sweet potato is superior in heat resistance and light stability compared to AP contained in other plants^36^. AP also interacts with both cellulose and pectin^39^. AP has traditionally been used as a natural food pigment.

In this research, we use *Paramecium* sp. for the screen of food pigments which can be used for DET. In addition, in order to compare how natural pigments act on cells, a comparison was made with the results using *Euglena* as a target cell^6^.

## Results

### Confirmation of reliable staining of dead cells with 10 food pigments and 2 synthetic dyes (Table 1)

**Table 1:** Types of pigments/dyes and staining.

	Natural food pigment										Synthetic dye	
	*Monascus*	Purple sweet potato	Yellow gardenia	Green gardenia^※^	Purple cabbage	Turmeric	Red beet	*Spirulina*	Kaoliang	Bamboo charcoal	Trypan blue	Methylene blue
MW	○	○	×	Δ	○	×	×	×	×	×	○	○
BC	○	○	×	×	○	×	×	×	×	×	×	○

*MW* microwave treated dead cells, *BC* Benzalkonium chloride treated dead cells.

○: All cells were stained within 1 min (30 cells or more was observed), and can be visually judged with 4 × objective lens.

Δ: It took 15 min to stain all cells.

× : Not all cells were stained within 15 min.

※: Mixture of yellow and blue gardenia.

Each pigment was dissolved in the dead cell suspension (MW or BC treatment). The time from the mixing of pigment until all the cells were stained was measured.

### Measurement of absorbance of single live or dead cells in the culture with natural food pigment or synthetic dye (Figs. 1, 2)

**Figure 1:**
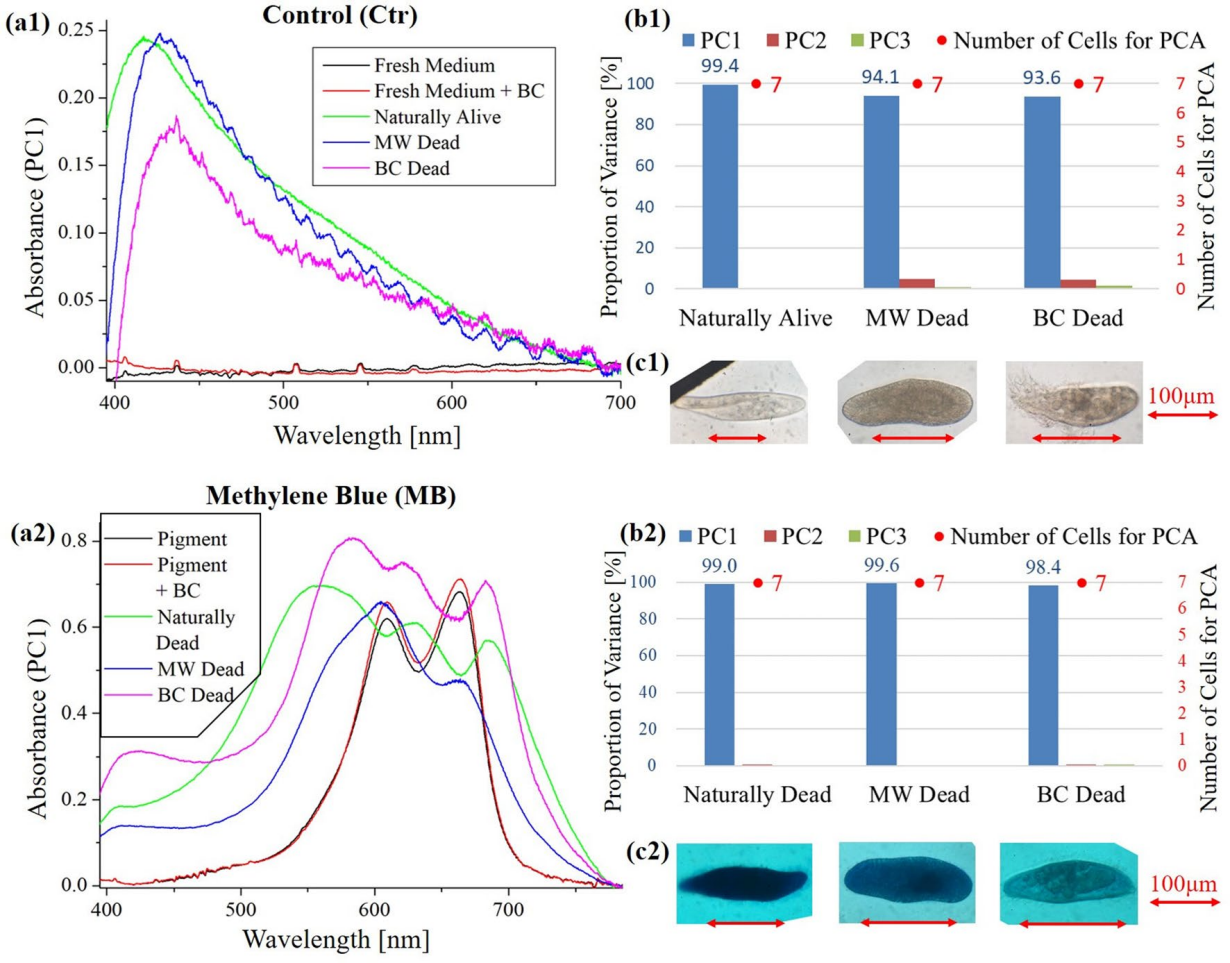
Principal component analysis (PCA) of absorption spectra of *Paramecium* in growth culture without pigment (Control sample) and with methylene blue. (**a**) First principal component (PC1) of absorption spectra of single cells (**b**) Proportion of variances of PC1 to PC3 in single cell absorption spectra and the numbers of cells for PCA (**c**) Bright field microscopic image of cells with the inverted microscope (IX71, OLYMPUS) with a 40 × objective lens. (a1)–(c1) Control sample (Ctr), (a2)–(c2) Methylene blue (MB). MW: Microwave treated dead cells, BC: Benzalkonium chloride treated dead cells.

**Figure 2:**
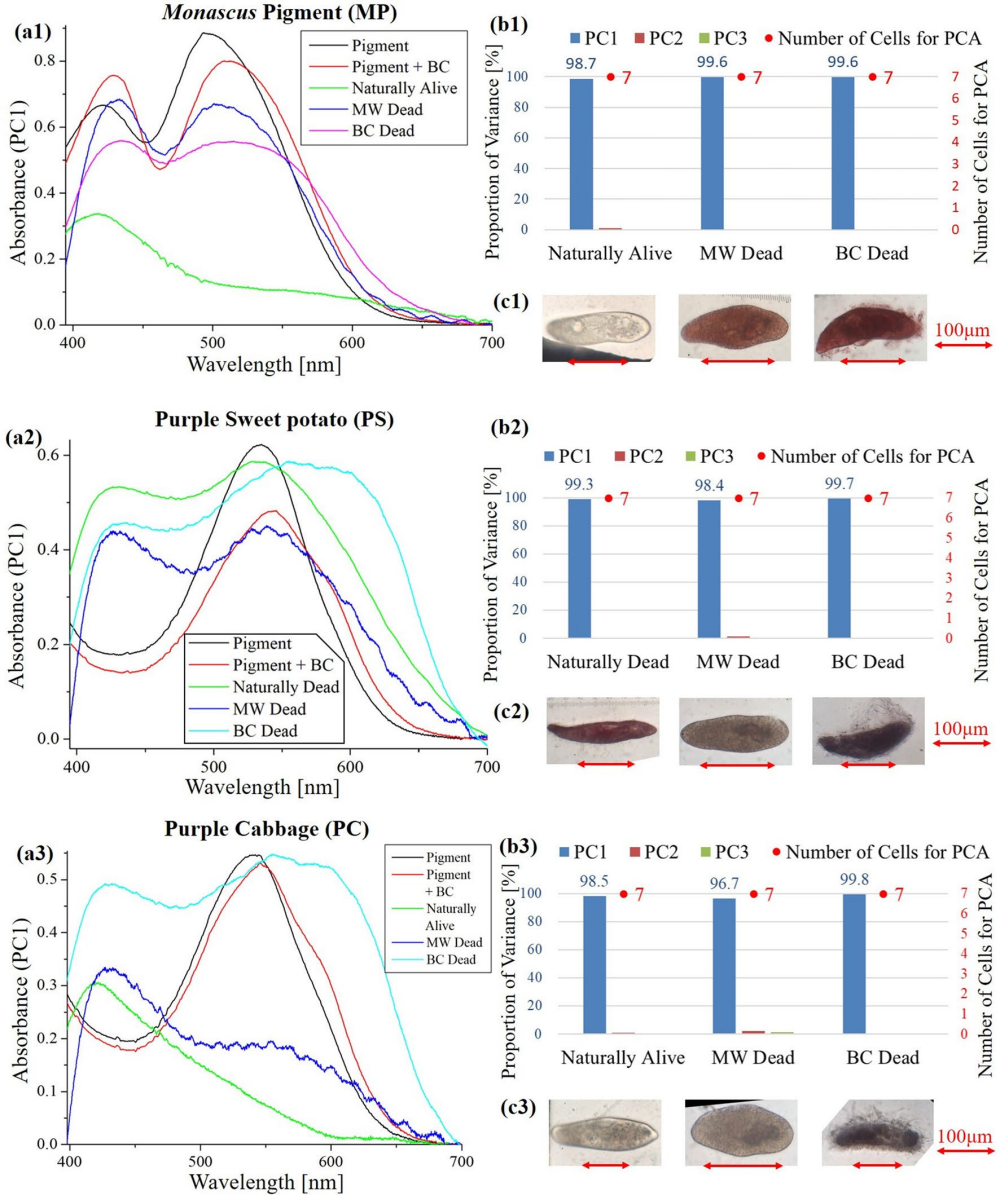
Principal component analysis (PCA) of absorption spectra of *Paramecium* in growth culture mixed with natural pigments. (**a**) First principal component (PC1) of absorption spectra of single cells (**b**) Proportion of variances of PC1 to PC3 in single cell absorption spectra and the numbers of cells for PCA (**c**) Bright field microscopic image of cells with the inverted microscope (IX71) with a 40 × objective lens. (a1)–(c1) *Monascus* pigment (MP), (a2)–(c2) Purple sweet potato (PS), (a3)–(c3) Purple Cabbage (PC). MW: Microwave treated dead cells, BC: Benzalkonium chloride treated dead cells.

Figures 1, 2 (a1 to a3) shows the first principal component (PC1) of absorption spectra of *Paramecium* cultured in growth medium (Table 4) with or without pigments. Since all the cells in the cell suspension mixed with MB and PS were stained, the absorbance spectrum of “Naturally alive” could not be measured.

**Table 2.** pH of culture mixed with pigment

	Ctr^※1^	MP	PS	PC	MB^※2^
Without 3 mM Tris	6.68	6.44	3.81	4.2	–
With 3 mM Tris	7.98	7.89	4.99	6.11	–

※1. Means “fresh growth medium” (Table 4)

※2. Unmeasured due to concern about dye staining on glass electrode of pH meter

*Ctr* control, *MP* *Monascus*, *PS* purple sweet potato, *PC* purple cabbage, *MB* Methylene blue

### Survival curve and ratio of dead cells of *Paramecium* in pigment mixed culture with or without Tris (Fig. 3)

**Figure 3:**
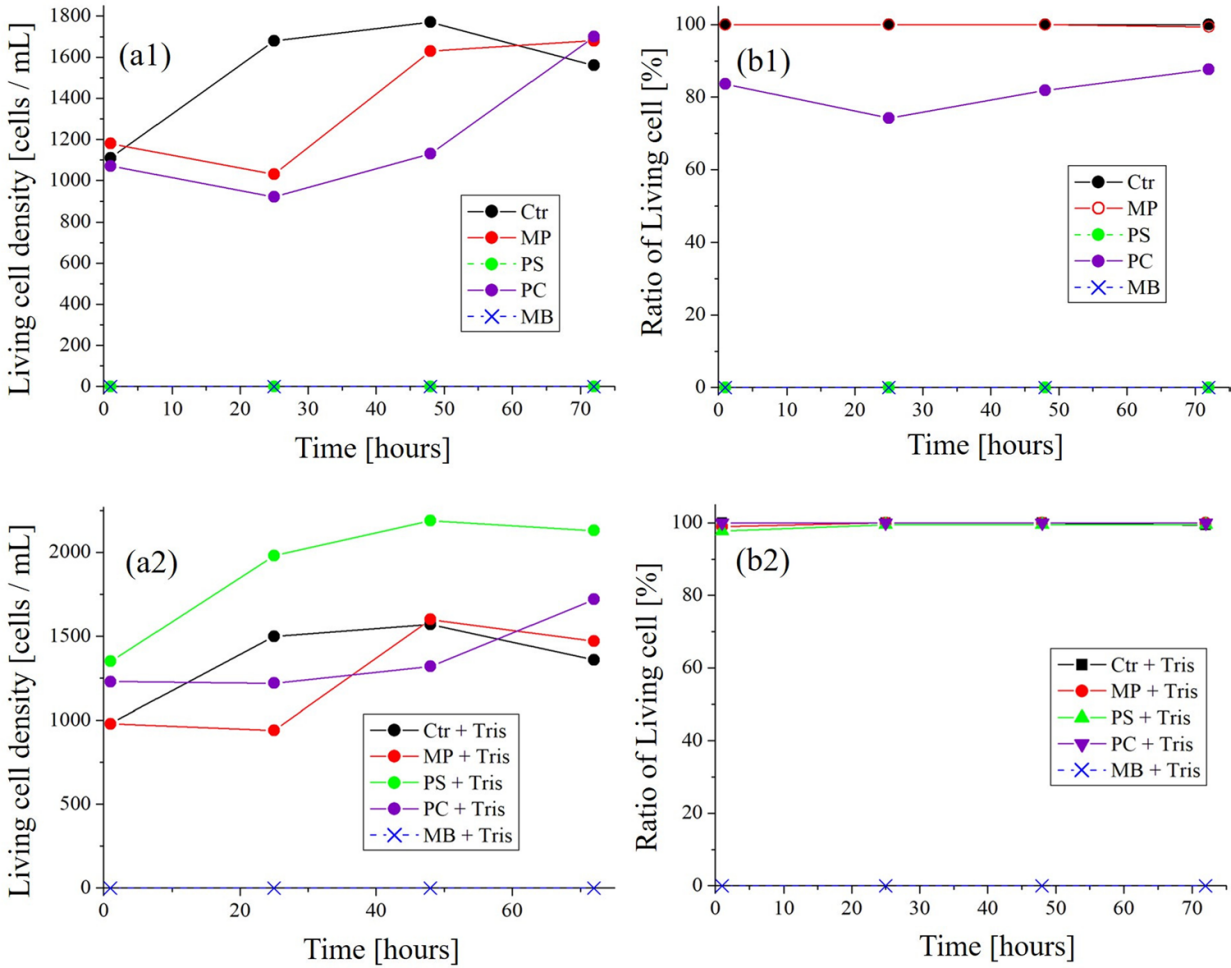
Survival curve and ratio of living cells of *Paramecium* in pigment mixed culture with or without Tris. (**a**) Survival curve of *Paramecium* in growth medium mixed with pigment (About “PS” and “MB” at 1 h from the beginning, because they have already been all dead and stained, subsequent living cell density was 0 cells/mL) (a1) without Tris (a2) with 3 mM Tris. (**b**) Ratio of living cells of *Paramecium* in growth medium mixed with pigment. (b1) without Tris (b2) with 3 mM Tris. *Ctr* Control, MP: *Monascus*, *PS *purple sweet potato, *PC* purple cabbage, *MB* methylene blue.

Figure 3 (a1,a2) shows the survival curve of *Paramecium* in growth medium mixed with or without Tris. Figure 3 (b1,b2) shows ratio of living cells mixed with or without Tris.

The results are based on a single measurement.

### pH measurement of growth medium mixed with pigment

The pH of control sample (Ctr) was close to the optimum pH of 7^40^. With anthocyanin pigments (PS and PC), the pH of the culture solution was markedly decreased. On the other hand, the pH of MP was slightly lower than that of Ctr, and did not show a large fluctuation like anthocyanin pigments.

The pH of each culture mixed with pigment and 3 mM Tris was about 1–2 higher than that without Tris.

### Comparison of *Paramecium* and *E. gracilis* for staining

Table 3 shows the differences in the staining, toxicity, and method of alleviation of toxicity for each pigment between *Paramecium* obtained in the present paper and *E. gracilis* in the preceding paper^6^.

**Table 3:** Comparison of *Paramecium* and *E. gracilis.*

	Staining			Toxicity			Alleviation of toxicity	
	MP	PS	MB	MP	PS	MB	PS	MB
*Paramecium*	○	○	○	Low	Extremely toxic	Extremely toxic	Tris addition	–
*E. gracilis*	○	○	Δ	Low	High	Middle	Glucose addition	Glucose addition

Staining: ○: In a sample treated with MW or BC Dead, all cells are stained within 3 min.

Δ: Although the above conditions are satisfied, it is difficult to distinguish colors from living cells.

Toxicity: Extremely toxic: All cells were stained within 1 h of pigment addition.

High: Inhibition of cell proliferation and increase in dead cells are observed.

Middle: Although growth is suppressed, the increase of dead cells within 2 days is within 10%

Alleviation of Toxicity”: How to improve survival.

*MW* microwave treated dead cells, *BC* Benzalkonium chloride treated dead cells, *MP* *Monascus*, *PS* purple sweet potato, *MB* methylene blue.

## Discussion

As summarized in Table 1, with the natural pigments of *Monascus* (MP), Purple sweet potato (PS), Purple cabbage (PC), and a synthetic dye of MB, dead cells treated with microwave (MW) or benzalkonium chloride (BC) of *Paramecium* were stained as vividly as to be clearly distinguished from live cells within 1 min. On the other hand, viability assay by green gardenia for cells treated with MW required 15 min until all cells were stained. In TB, cells treated with MW were stained, but cells treated with BC were not stained. This is because the reaction of TB with BC resulted in the formation of blue precipitates that prevented TB from penetrating into the cells. With the other pigments, by contrast, dead cells were not stained clearly enough to be visually distinguished. Therefore, in this study, experiments were conducted on the above four types of pigments (MP, PS, PC, and MB). Generally, natural pigments distributed in Japan are not composed of a single component, but are composed of various compounds. Further, the composition is likely to differ depending on each manufacturing company. However, as the food pigments (MP, PS) have passed the component standards (species of raw materials, color value, wavelength range of maximum absorption) as stated in the JSFA^35^, there is no significant variation in quality. The absorption maximum of MP is 493 nm (Fig. 2, a1). This actually satisfies the JSFA standard (maximum absorption part in the wavelength of 480–520 nm), and it is suggested that monascorubrins which show red color are the main components^32^. Also, since PC is a pH determining reagent, it is manufactured so that the concentration becomes appropriate when a predetermined amount is dissolved in an aqueous solution^41^.

It was confirmed for the first time that the metal mesh cell capture (MMCC) method can be applied to absorption spectroscopic imaging instead of the Lab-on-a-chip conventionally used as a confined space for capturing live cells. In the measurement (Figs. 1, 2), since the proportion of variances of each sample are approximately 1, the spectrum of each sample can be represented by PC1 (Figs. 1, 2b). It shows that all the samples of dead cells were stained evenly by each natural pigment or a synthetic dye. In the microscopic observation (40 × objective lens), one can readily distinguish between the control sample of “Naturally Alive” in Fig. 1(c1) and other samples of stained dead cells (Figs. 1c2, 2c1–c3), even at low magnification (4 × objective lens). However, when “MW Dead” of PS and PC was diluted 10 times with water, the staining became slightly lighter (Figs. 2c2, c3). It is difficult to distinguish between “Naturally Alive” and unstained dead cells (“MW Dead”, “BC Dead”) at low magnification (4 × objective lens) in the control sample (Fig. 1, c1). In addition, since the living cells in the natural pigment solution were not stained (Figs. 2c1, c3), it was found that these natural pigments can be applied to DET.

From Fig. 3, it was confirmed that the natural pigments do not show marked growth inhibition or toxicity to living cells as compared with the synthetic dyes (MB). For this performance, however, addition of Tris was required for PS and PC that made the cultures more acidic than the range of viable pH. When the pH is 5 or more, the survival rate was confirmed to be almost 100%. It was found that the stained dead cells can be monitored for the cell viability assay for 3 days. In addition, their cost is lower than the methods that require expensive equipment, and they are safe for the environment.

MP is known to exhibit anti-bacterial activity. Its target cells are diverse such as gram-positive and gram-negative bacteria, yeast, and filamentous fungi^34,42^. As shown in Fig. 3, however, it is most likely that *Paramecium* is not affected by anti-bacterial activity by MP. On the other hand, when PS and PC were added, ratio of dead cells increased over time as shown in Fig. 3. PS and PC in Fig. 3 (b1) seems to indicate anti-microbial activity. Actually, it is known that anthocyanin suppresses gram-negative bacteria but not gram-positive bacteria^38^. However, it is also suggested that the addition of anthocyanin food pigment (PS and PC) caused a decrease in the survival rate, but it was improved by the addition of Tris. This suggests that the addition of dye lowered the viability of *Paramecium* by lowering the pH of the culture solution below the viable range. When the pH was 5 or more, the survival rate was confirmed to be almost 100% (Fig. 3, b2 and Table 2). The optimum pH for *Paramecium* is around 7^40^. Therefore, the harmful effect of anthocyanin food pigments (PS and PC) on *Paramecium* was mainly a decrease in pH, suggesting that the pigment itself does not act directly. By contrast, in the unicellular microalgae *Euglena*, if the final concentration of CM medium (standard culture of *Euglena*: pH 3.5 before Tris addition)^43^ is adjusted to 1% of PS and 3 mM of Tris, respectively, although the pH of the culture solution was 3.7 within the viable range, the number of dead cells increased (data not shown). Instead, for *E. gracilis*, the addition of glucose together with PS has been shown to improve survival^6^. Therefore, in the case of *E. gracilis*, it is considered that dead cells increased due to mechanism of PS acting on cells different from *Paramecium*. In addition, despite the low toxicity of MB to *Euglena*^6^, the toxicity of MB in *Paramecium* was extremely high (Fig. 3).

**Table 4:** Composition of growth medium for *Paramecium.*

Composition (14 mL)	Volume (mL)
0.05% (W/V) Wakamoto solution^※1^	10
Suspension of *Paramecium*	3
Suspension of *C. reinhardtii*^※2^	1

※1. Tablet gastrointestinal drug “Wakamoto” was dissolved in purified water.

※2. *C. reinhardtii* was cultured in TAP (Tris–Acetate-Phosphate) medium to stationary phase at the same place as sample of *Paramecium*. TAP medium is a standard culture of *Chlamydomonas*^45^.

Since MP shows almost no toxicity in both cells and a clear staining was obtained, it can be said that MP is the most excellent reagent of viability assay among the natural pigments examined. MB was extremely toxic to *Paramecium* but not very toxic to *E. gracilis*. It is known that PS, PC, MB and TB do not fade by light, but MP fades in about 4 days^6^. On the other hand, TB reacts with BC to form insoluble crystals, so it cannot be applied to DET with BC (Table 1).

From the above results, it is important to select the pigment properly and appropriate auxiliary reagents to protect cells. It will be a challenge in the future to search for the optimal use conditions according to each purpose because natural pigments have a wide range of applications due to many variations. The DET method with natural pigments can play an important role in the fields of food, hygiene, and life sciences that require safety from daily environments such as cafeterias and families to special facilities such as laboratories and factories. Furthermore, a method for viability assay by flow cytometry using an edible synthetic colorant has been established^22^, and there are examples of using natural food pigments or edible synthetic colorant as vital dyes (MP and RC are not listed in the literature)^44^. Hence, the fields are broader where the natural food pigments found in this study can be used as DET.

## Methods

### Sample preparation

Samples were prepared for each experiment as follows. The *Paramecium sp.* was obtained from YYD Co., Ltd., and cultured with *Chlamydomonas reinhardtii* (NIES-2238) in solution of gastrointestinal drug “Wakamoto” consisting of natural ingredients derived from digestive enzymes, lactic acid bacteria and brewer's yeast (WAKAMOTO PHARMACEUTICAL CO., LTD.). Composition of growth medium for *Paramecium* is shown in Table 4. Each culture was stationary and aerobically under continuous illumination with a cool white fluorescent light at 80 to100 μmol/m^2^/s and at constant temperature 28 °C. The incubation period and initial cell density of *Paramecium* for each experiment are shown in Table 5.

**Table 5:** Incubation period and initial cell density of *Paramecium.*

Experimental item	Table/figure number	Incubation period (day)	Initial cell density (cell/mL)
Stain confirmation	Table 1	24	1,010
Naturally alive (ASI)	Figures 1 and 2(a1, a3)	29	1,210
Naturally dead (ASI)	Figures 1(a2), 2 (a2),	29	1,210
MW dead (ASI)	Figures 1 and 2 (a)	10	435 (the 9th day)
BC dead (ASI)	Figures 1 and 2 (a)	11	435 (the 9th day)
Survival curve	Figure 3(a1, a2)	11	1,157
Living ratio	Figure 3(b1, b2)	11	1,157

*ASI* Absorbance spectral imaging, *MW* microwave treated dead cells, *BC* Benzalkonium chloride treated dead cells.

### Preparation of dead cells of *Paramecium*

Dead cells were obtained by the following two treatments. Cell suspension was treated with microwave at 2.45 GHz until it boiled (MW Dead). 10%(W/V) benzalkonium chloride (BC) solution (NIHON PHARMACEUTICAL CO., LTD) was added to each cell suspension to a final concentration of 0.2% (BC Dead). Since the BC solution is transparent, it does not itself stain cells.

### Adjustment of pigment concentration of sample

The pigment was added to the cell suspension of *Paramecium* and mixed. The pigment concentration of the sample in each experiment is shown in Table 6.

**Table 6:** Pigment/dye concentration in cell suspension in each experiment.

Pigment/dye	Pigment concentration %(W/V)	Related table/figure
Control (Ctr)	0	Tables 1 and 2, Figs. 1, 2 and 3
*Monascus* (MP)	1	Tables 1 and 2, Figs. 1, 2 and 3
Purple sweet potato (PS)	1	Tables 1 and 2, Figs. 1, 2 and 3
Yellow gardenia	1	Table 1
Green gardenia^※^	1	Table 1
Purple cabbage (PC)	1	Tables 1 and 2, Figs. 1, 2 and 3
Turmeric	1	Table 1
Red beet	1	Table 1
*Spirulina*	1	Table 1
Kaoliang	1	Table 1
Bamboo charcoal	1	Table 1
Trypan blue	0.05	Table 1
Methylene blue (MB)	0.03	Figure 3
Methylene blue (MB)	0.05	Table 1, Figs. 1 and 2

※Mixture of Yellow and blue gardenia.

The pigments and producers used in this study are as follows.

- Purple cabbage powder (Universe of Science, Inc.)
- Turmeric (GABAN Co., Ltd.)
- Trypan blue (TOKYO CHYEMI-CAL INDUSTRY Co., Ltd.)
- Methylene blue (KOKUSAN CHEMICAL Co., Ltd.)
- The others (Watashinodaidokoro Co., Ltd.)

Only purple cabbage is a pH determining reagent, and other pigments are edible natural pigments.

### Observation of stained cells by bright field microscope

The bright field microscopes and objective lenses used in each experiment are as follows^6^ (Tables 7, 8).

- Measurement of absorbance of single living or dead cells (Figs. 1, 2)
  Inverted research microscope (IX71, OLYMPUS) with the 40 × or 100 × objective lens.
- Other experiments.
  Digital biological microscope (GR-D8T2, Shodensha, Inc.) with the 4 × or 10 × objective lens.

**Table 7:** Conditions for microscopic observation (cell observation and absorption spectral imaging).

Experimental item	Table/fig. number	Dilution rate	Cells container for observation	Microscope	Objective lens^※^	Photon flux density (µmol/m^2^/s)	Illumination region (mm)	Exposure time (s)
Stain confirmation	Table 1	1	Glass bottom dish	GR-D8T2	4	14	Φ4.5	–
Naturally alive (ASI)	Figures 1 and 2 (a1, a3)	1	Metal mesh cell capture	IX71	40	1,900	Φ3	0.15
Naturally dead (ASI)	Figures 1(a2), 2(a2)	1	Metal mesh cell capture	IX71	100	1,900	Φ3	0.2
MW dead (ASI)	Figures 1 and 2 (a)	10	Glass bottom dish	IX71	100	1,950	Φ3	0.5
BC dead (ASI)	Figures 1 and 2 (a)	10	Glass bottom dish	IX71	100	1,950	Φ3	0.5
Survival curve	Figure 3	1	Plankton counter plate	GR-D8T2	10	14	Φ4.5	–
Ratio of living cell	Figure 3	1	Plankton counter plate	GR-D8T2	10	14	Φ4.5	–

※ Magnification of the objective lens: Details of the objective lenses are shown in the following table (Table 8).

*ASI* Absorbance spectral imaging, *MW* microwave treated dead cells, *BC* Benzalkonium chloride treated dead cells.

**Table 8:** Specifications of objective lenses.

Magnification	NA	Model
4	0.1	GR-D8T2 (Shodensha)
10	0.25	GR-D8T2 (Shodensha)
40	0.55	SLCPlanFLI (OLYMPUS)
100	0.85	LCPlanFLN (OLYMPUS)

### Measurement of cell density

Cell counting was performed using plankton counter plates (MPC-200, Matsunami Glass Ind., Ltd.). To fix the cells, 10%(W/V) benzalkonium chloride (BC) solution was added to each sample to a final concentration of 0.2%. For counting, a bright field microscope (GR-D8T2) equipped with a 10 × objective and a plankton calculator plate were used. The number of cells in the 20 × 20 section of the plankton counter plate was counted (Tables 5, 8). The grid pitch of one section of the plankton counter plate is 500 μm.

### Confirmation of reliable staining of dead cells with 10 food pigments and 2 synthetic dyes (Table 1)

#### Sample preparation

Cells were cultured to a cell density 1,010 cells/mL for 24 days (Table 5). Cell suspensions were treated with MW or BC. 10 kinds of natural pigments were dissolved in the dead cell suspension to be adjusted to a final concentration of 1% (W/V) and thoroughly mixed by tapping. Similarly, two types of synthetic dyes were dissolved in dead cell suspension and adjusted to a concentration of 0.05% (Table 6).

### Confirmation of stained dead cells and measurement of staining time

Immediately after mixing the pigment, cells in a glass bottom dish (D11130H, Matsunami Glass Ind., Ltd.) were observed with the bright field microscope (GR-D8T2) (Table 7). The time from the mixing of pigment until all the cells (above 30 cells) were stained in the observation area was measured. The results are shown in Table 1.

### Measurement of absorbance spectra of single live or dead cells in the culture with natural food pigments or synthetic dyes (Figs. 1, 2).

#### Sample preparation for measurement of absorbance spectral imaging of single cells

Cell suspensions were divided into the following three kinds of samples^6^.

1. Naturally Alive/Dead: Spontaneously dead cells in normal culture (growth medium)
  Cells were measured by absorbance spectral imaging after incubation for 2 days with pigment.
2. MW Dead: Dead cells treated with microwave
  After treatment of microwave, pigment was added and absorbance imaging was measured.
3. BC Dead: Dead cells treated with 0.2%(W/V) benzalkonium chloride (BC)

After treatment of BC, pigment was added and absorbance imaging was measured. Since the BC solution is transparent, it does not contribute to staining of cells.

On measurement of absorbance spectral imaging, each sample was diluted tenfold with growth medium for *Paramecium* and injected into a glass bottom dish or Metal Mesh Cell Capture (MMCC). As a control sample, pigment solutions were prepared with fresh growth medium for *Paramecium* as solvent (Table 7).

- Fresh Medium: Pigment solution in which the solvent is fresh growth medium for *Paramecium*
- Fresh Medium + BC: Pigment solution in which the solvent is fresh growth medium for *Paramecium* with 0.2% BC

Control samples were injected into a glass bottom dish without dilution. Then, samples were measured by scan-free, non-invasive absorbance spectral imaging *A*(*x, y, λ*) microscopy^46^ (Details are described below).

### Measurement method of single-cell absorbance spectral imaging

**Figure 4:**
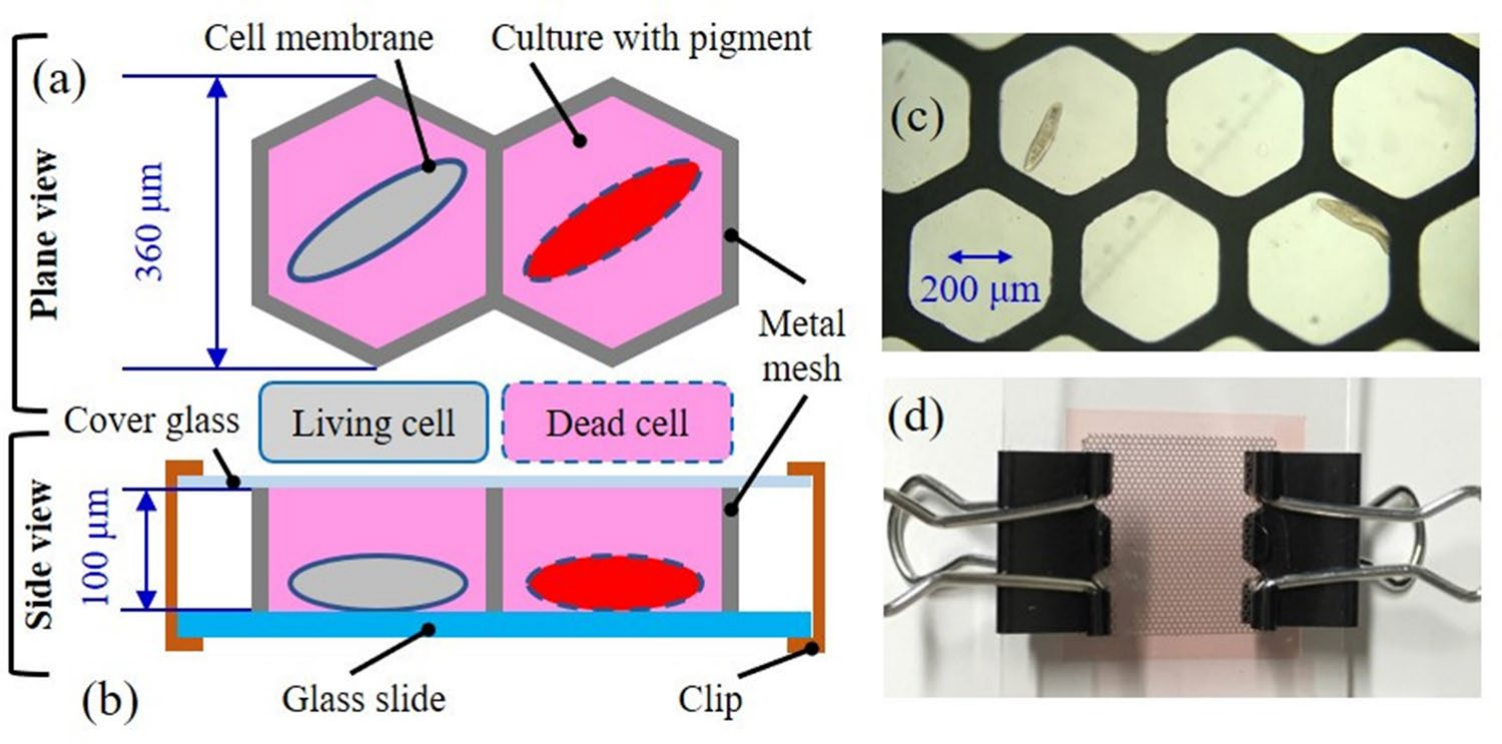
Conceptual diagram of dye exclusion test and Metal Mesh Cell Capture (MMCC). (**a**) Plane view of MMCC, (**b**) side view of MMCC, (**c**) bright field microscopic image of cells with the inverted microscope with the 10 × objective lens (NA 0.30, UPlanFLI, OLYMPUS), (**d**) appearance of MMCC.

Detailed method of scan-free absorbance spectral imaging was previously described^46^. The measurement method used in this study was the same as in the preceding paper^6^, except that the sample was replaced from *Euglena* with *Paramecium*. Only the differences are described below. The glass bottom dish or MMCC (Metal Mesh Cell Capture: See Fig. 4) with sample was set on the inverted microscope and observed with the 40 × or 100 × objective lenses from below. The light source was a 150 W Xenon lamp (Hamamatsu) to illuminate a region of 3 mm in diameter of the sample from above through a condenser. The intensity (photon flux density) on the sample was 1,900–1,950 μmol/m^2^/s for 0.15, 0.2 or 0.5 s exposure (Table 7). The transmitted light was transferred through the objective and a focusing lens to the side port of the microscope.

### Method of metal mesh cell capture (MMCC)

Modeling mesh 23 (HASEGAWA CORPORATION) is a regular hexagonal metal mesh with a side of 180 μm and a thickness of 100 μm. The metal mesh was placed on a slide glass, and the cell suspension was filled into the regular hexagonal space of the mesh such that air bubbles did not enter. Furthermore, a cover glass is placed on the metal mesh and fixed with clips from both sides, so that cells can be trapped in regular hexagonal spaces (Fig. 4).

Compared to Lab-on-a-chip with the microwells^47^, which is a device used in the conventional method for capturing live cells, this method is characterized by the ability to easily fabricate a low-cost and robust cell capture. In addition, Modeling mesh of various shapes and sizes is available, and various cell capture can be selected. This paper is the first report of a single cell capture method using MMCC.

### Principal component analysis (PCA) of absorption spectra of single cells

Absorption spectra obtained from different single cells in each sample were principal component analyzed. The spectra of the first principal component (PC1), the proportion of variances of PC1 to PC3, and the number of cells for PCA are shown in Figs. 1 and 2. For the PCA, the free software for scientific data analysis “Past (Ver. 3.22)” was used^48^.

### Survival curve and ratio of living cells of *Paramecium* in culture mixed with pigment (Fig. 3)

#### Sample preparation for the survival curve and the ratio of living cells

Cells were cultured to a cell density 1157cells/mL for 11 days (Table 5). The suspension was mixed with pigment. The pigment concentration of each sample is shown in Table 6. In the Tris (Trimethylolaminomethane)-added sample, the concentration of Tris was adjusted to a concentration of 3 mM. The cells were counted once a day and left aerobically in the dark for other periods.

### Cell counting method for the survival curve and ratio of living cells (Fig. 3)

The count for the survival curve was performed using plankton counter plates (MPC-200, Matsunami Glass Ind., Ltd.). In order to stop the movement of *Paramecium* during counting, it is necessary to fix the cells by adding BC solution. However, since the cells fixed by the addition of the BC solution are immediately stained, they cannot be distinguished from the naturally dead cells (cells that were dead in the cell suspension before the addition of the BC solution). Therefore, first, stained cells contained in the cell suspension (Naturally Dead) were counted (N_ND_). N_ND_ means the number of naturally dead cells in the cell suspension. Next, 10% (W/V) BC solution was added to the same cell suspension to a concentration of 0.2% (the volume of 10% BC solution added to the cell suspension was only 2%). The number of cells contained in the sample in which the cells were fixed was counted (N_All_). N_All_ means the total number of cells in the cell suspension. From the above, the living cell density was calculated by subtracting N_ND_ from N_All_ as the number of living cells in the cell suspension. If “N_ND_ > N_All_” due to measurement error, the living cell density was recorded as 0 cells/mL. For the counting, a bright field microscope (GR-D8T2) with the 10 × objective lenses were used (Tables 7, 8). The counting was performed once a day for 4 days. The results are shown in Fig. 3. The survival rate R_Living_ % was calculated as “(N_All_ − N_ND_)/N_All_ × 100”. When “N_ND_ > N_All_”, R_Living_ = 0%. The results are shown in Fig. 3. The culture with or without BC was separately prepared from the same culture. The dead cell density can be estimated from survival curve and ratio of living cells. The results are based on a single measurement.

### pH measurement of growth medium mixed with pigment

A glass-electrode type hydrogen ion concentration indicator (D-55: HORIBA, Ltd.) to measure the pH of culture was used. The solvent of each culture mixed with pigment was fresh growth medium (Table 4), and the pH of the sample in which only the pigment was mixed and that in which the pigment and 3 mM Tris were mixed were measured. The concentration of each pigments was adjusted to 1% (Table 6). However, the pH of MB was not measured because there was a risk of MB strongly staining the glass electrode.
